# Prevalence, Knowledge, and Attitude Toward Substance Abuse, Alcohol Intake, and Smoking Among Male High School Students in Riyadh, Saudi Arabia

**DOI:** 10.7759/cureus.33457

**Published:** 2023-01-06

**Authors:** Ibrahim Alenazi, Abdulkarem Alanazi, Mohammed Alabdali, Abdulaziz Alanazi, Salam Alanazi

**Affiliations:** 1 Family Medicine, Prince Mohammed bin Abdulaziz Hospital, Riyadh, SAU; 2 Medicine, King Saud bin Abdulaziz University for Health Sciences (KSAU-HS), Riyadh, SAU

**Keywords:** prevalence, young students, drug abuse, alcohol, smoking

## Abstract

Background: The prevalence of smoking, alcohol intake and drug use among young people is increasing worldwide.

Aim: The aim is to determine the prevalence, knowledge, and attitudes of male high school students toward substance abuse, alcohol intake, and smoking in Riyadh, Saudi Arabia.

Methods: A survey was conducted from March to May 2021 using a self-administered questionnaire distributed to male high school students in grades 10 to 12 from randomly selected eight public and three private schools in Riyadh, Saudi Arabia.

Results: A total of 400 male high school students participated in this study. The mean age of participants was 17.5 ± 1.3 years (range: 15 to 21 years old). A total of 281 students (70.2%) attended eight public/government schools and 119 (29.8%) attended three private or international schools. Most students (>70%) had knowledge of the harmful effects of smoking, alcohol, and drugs. Nonetheless, the prevalence of smoking, alcohol intake, and drug abuse was 27.8%, 11.5%, and 9.5%, respectively. Students began smoking before age 15, drinking alcohol before age 20, and using drugs as early as age 14. Most smokers and students that drank alcohol procure these substances by themselves whereas many students that took illegal drugs from friends. These substances markedly affected the students' school performance.

Conclusion: The prevalence of smoking, alcohol intake, and drug use were high. Students began smoking, drinking alcohol, and using abused drugs at an early age, which were influenced by friends, peers, or their siblings. Some students purchase these substances by themselves while some got them from friends, especially alcohol. These practices affected their performance at school. Although many students were aware of the harmful effects of smoking, alcohol intake, and substance abuse, some students had opposing perspectives. Therefore, health authorities need to educate these students and institute structural and emotional support for students who are in these vices to mitigate misuse, long-term use, and addiction.

## Introduction

Substance abuse (including alcohol and other illicit drugs) and tobacco smoking (including vaping, hookah, and e-cigarettes) usually commence during the teenage years [1]. In recent years, substance abuse and tobacco smoking have become more prevalent among youths; however, this practice is unsafe, particularly for individuals of this age [1,2].

The 2011 to 2014 data from the CDC and the National Food and Drug Administration of the United States indicated that an estimated 4.6 million middle and high school students use a tobacco-based product, of which 2.2 million of these students use more than two tobacco products at one time [2]. In another report published in 2016, the estimated number of tobacco users in US high school students increased to 4.7 million [3]. A survey conducted among high school and middle school students in the US in 2016 revealed that 47.2% of high school and 42.4% of middle school students use more than two tobacco products [4].

A high prevalence of cigarette smoking and shisha use among students aged 13-15 years has been reported by Co-operation Council for the Arab States of the Gulf (GCC) member states [5]. High susceptibility to initiating smoking was reported to be troubling, with boys more likely to smoke than girls (5). A study conducted in 2006 among Saudi medical students revealed a prevalence of 13% active smokers, with shisha being the most commonly smoked product (44.1%), followed by cigarettes (32.2%). Another study conducted among Saudi adolescents in 2010 revealed a tobacco smoking prevalence of 9.72% (12.43% among boys and 6.65% among girls) [6]. In contrast, the reported prevalence of substance abuse among male secondary school students in Saudi Arabia was 8.8%, whereas that of male secondary students that drank alcohol was 9.3% (7). The most common illicit drug used by students was cannabis (51.4%), followed by glue/solvents (48.6%) and amphetamine (45.7%) [7].

A report from Saudi Arabia showed that students from higher-income families that have larger daily allowances, spend less time studying, skip classes more frequently, are less religious, spend more time at home, and drink more soft drinks were found to be more likely to smoke and use illicit substances [6]. Another significant predictor of smoking and substance abuse among youths is having friends who smoke [8]. A study revealed that 82.4% of students are sufficiently aware of smoking and substance abuse, and this is significantly correlated with having educated parents, family income, and parents that live together [9].

Furthermore, studies conducted among Saudi high school students revealed that drug abuse and smoking are highly prevalent and estimates of existing knowledge and awareness vary [10]. However, reports on substance abuse and tobacco use among adolescents in Saudi Arabia have reported mixed results depending on the geographical location [6-10]. With the reported high frequencies of smoking and use of illicit substances among high school students and adolescents, it is imperative to assess their knowledge, attitudes, and practices regarding substance abuse and tobacco smoking to understand the reasons for the high prevalence of such practices. Therefore, this study was carried out to determine the prevalence of substance abuse and smoking among high school students, and their knowledge and attitude toward substance abuse and smoking to address this issue.

## Materials and methods

We conducted a cross-sectional survey in March, April, and May 2021 using a self-administered questionnaire distributed to male high school students in grades 10 to 12 at eight public and three private schools in Riyadh, Saudi Arabia. School selection was performed using a random sampling technique. A random drawing of lots from a list of all male schools in Riyadh, Saudi Arabia, was performed via a random selection of 11 high schools in Riyadh, Saudi Arabia. The necessary permits were secured from each selected school. 

The sample size was calculated using the formula: n = Z 2p (1 - p)/d 2, where n is the sample size, z is the level of confidence (95% or 1.96), p is the expected proportion in the population (60%), and d is the absolute error or precision (5%). Assuming that the prevalence of smoking and substance abuse was approximately 15%, the calculated required sample size was 369 students. We collected data from up to 400 male students to account for non-participation and low or incomplete responses. 

The self-administered questionnaire was developed using constructs from previous related literature [5-9] in the English language that were translated into Arabic. The questionnaire included the demographic characteristics of the students, social influences, attitudes, and knowledge. A pilot study was conducted among 20 students to verify the validity of the questionnaire. This pilot study included one male class from public high schools in Riyadh that was not part of the study sample. The questionnaire was modified following a thorough review of the results of the pilot study. Cronbach’s alpha for the pilot study was 0.86.

Data were analyzed using the Statistical Package for Social Sciences (SPSS) version 23.0 (SPSS Inc., IBM, Armonk, NY, USA). Descriptive statistics of the survey variables were computed as means and frequencies. Chi-square and t-tests were performed to determine the significant differences between categorical variables. A Pearson correlation test was performed to determine the bivariate relationship between the two variables. Statistical significance was set at p < 0.05. 

To ensure the confidentiality and anonymity of our participants, the data collection tool did not include the participants’ identifiers (name and address) or telephone numbers. Participation was voluntary, and there were no remuneration or payments for participation in the survey. Students were informed of the study objectives, and their participation was voluntary. Ethical approval to conduct the study was obtained from the Institutional Review Board of the Ministry of Health (Cluster 2), Riyadh, Saudi Arabia (IRB00018774), and the Ministry of Education, Saudi Arabia (transaction number 22421).

## Results

​A total of 400 male high school students participated in this study. The mean age was 17.5 ± 1.3 years (range: 15 to 21 years old). A total of 281 students (70.2%) attended a public/government school and 119 (29.8%) attended a private or international school. Table 1 shows the demographic profiles of the participants.

**Table 1:** Demographic characteristics of the 400 male high school students who participated in the survey.

Demographic variables	Mean ± SD (range)	n (%)
Age in years	17.5 ± 1.3 (15 – 21)	
Grades of the last year	2.7 ± 0.6 (1.0 – 3.0)	
School		
Public / Government		281 (70.2)
Private / International		119 (29.8)
School level		
First		35 (8.8)
Second		92 (23.0)
Third		273 (68.3)
Grades of the last year		
Good		21 (5.3)
Very good		89 (22.3)
Excellent		290 (72.5)
Father’s educational level		
Illiterate		17 (4.3)
Primary		23 (5.8)
Intermediate		28 (7.0)
Secondary		109 (27.3)
University		156 (39.0)
Postgraduate		67 (16.8)
Mother’s educational level		
Illiterate		30 (7.5)
Primary		38 (9.5)
Intermediate		41 (10.3)
Secondary		83 (20.8)
University		180 (45.0)
Postgraduate		28 (7.0)
Father’s employment status		
Unemployed / retired		155 (38.8)
Employed		245 (61.3)
Mother’s employment status		
Unemployed / retired		254 (63.5)
Employed		145 (36.5)
Family’s monthly income, in SAR		
<5,000		86 (21.5)
5,000-15,000		49 (12.3)
15,001-20,000		111 (27.8)
>20,000		72 (18.0)
Do not know		82 (20.5)
Total number of brothers and sisters	6.8 ± 2.3 (0 - 15)	
Daily pocket money allowance to school, in SAR		
<10		235 (58.8)
10 – 14		144 (36.0)
15 and more		21 (5.3)

Smoking

A total of 134 students (27.8%) were smokers. Of these students, 81 (20.3%) smoked cigarettes while 53 smoked a combination of cigarettes, electronic vapes, hookahs, pipes, cigars, and khat/kyat (13.3%). Of the 134 students who smoked, 79 (58.9%) began smoking before age 15. One hundred and two students (76.1%) could buy the product smoked from nearby stores without assistance. One hundred and twenty of the 134 students (89.6%) claimed to smoke on a daily basis, while 55 students (41.0%) claimed to smoke at least half a pack of cigarettes per day.

There were no significant differences in the age between smokers and non-smokers (smokers: 17.6 ± 1.4 years versus non-smokers 17.4 ± 1.3 years, p=0.312). There were also no significant differences in the proportion of smokers and non-smokers as to the father's educational level (p=0.316), mother's educational level (p=0.321), family monthly income (p=0.729), father's employment status (p=0.957) and mother's employment status (p=0.522). Smoking was more common among students who had more than five siblings than among those who had fewer than five siblings (n=115, 35.5% versus 19, 25.0%, p=0.081). Smoking was not significantly correlated with the number of siblings (r=0.087, p=0.081), school level (r=-0.014, p=0.777), age (r=0.051, p=0.312), grades in the previous year (r=-0.057, p=0.257), father’s educational level (r=-0.050, p=0.317), mother’s educational level (r=-0.050, p=0.322), father’s employment (r=-0.001, p=0.987), mother’s employment (r=-0.032, p=0.523), and family monthly income (r=-0.017, p=0.730) (Table 2).

**Table 2:** Smokers versus non-smokers according to demographic variables

Variables	Smoker n=134	Non-smoker n=266	p values	Correlation (p values)
Age in years, mean (SD)	17.6 (1.4)	17.4 (1.3)	0.312	0.051 (0.312)
Father’s education, %				
Secondary or less	64 (36.2)	113 (63.8)	0.316	-0.050 (0.317)
College and above	70 (31.4)	153 (68.6)		
Mother’s education, %				
Secondary or less	69 (35.9)	123 (64.1)	0.321	-0.050 (0.322)
College and above	65 (31.3)	143 (68.8)		
Family income, %				
15,000 SAR or less	84 (34.1)	162 (65.9)	0.366	-0.017 (0.730)
More than 15,000 SAR	50 (32.5)	104 (67.5)		
Father’s employment, %				
Employed	82 (33.5)	163 (66.5)	0.987	-0.001 (0.987)
Unemployed	52 (33.5)	103 (66.5)		
Mother’s employment, %				
Employed	46 (31.5)	100 (68.5)	0.522	-0.032 (0.523)
Unemployed	88 (34.6)	166 (65.4)		
Number of siblings, %				
Less than five	19 (25.0)	57 (75.0)	0.081	0.087 (0.081)
Five and more	115 (35.5)	209 (64.5)		
Grade level				
Good	11 (52.4)	10 (47.6)	0.165	-0.057 (0.257)
Very good	28 (31.5)	61 (68.5)		
Excellent	95 (32.8)	195 (67.2)		
School level				
First	13 (37.1)	22 (62.9)	0.885	-0.014 (0.777)
Second	30 (32.6)	62 (67.4)		
Third	91 (33.3)	182 (66.7)		

Alcohol

A total of 46 students (11.5%) claimed they drank alcohol that was mainly locally made, such as Arak and others (n=29 of 46, 63.0%). Thirty of the 46 students (65.2%) began drinking alcohol before age 20. Twenty-eight of the 46 students (60.9%) obtained these alcoholic drinks by themselves. Thirty-nine of the 46 students (84.8%) consumed 1-3 bottles per day.

There were no significant differences in the age between students that consume alcohol and those that do not consume alcohol (17.5 ± 1.5 years versus 17.5 ± 1.3 years, p=0.701). There were significantly more students who belonged to those with family income of more than 15,000 SAR a month who drank alcohol compared to those with family income of <15,000 SAR a month (p=0.008). A few students who drank alcohol had excellent grades (p=0.029). ​Alcohol drinking was significantly negatively correlated with grades in the previous year (r=-0.109, p=0.029) and positively correlated with higher family income (r=0134, p=0.008) (Table 3).

**Table 3:** Alcohol versus non-alcohol drinkers according to demographic variables

Variables	Alcohol drinker n=46	Non-alcohol drinker n=354	P values	Correlation (p values)
Age in years, mean (SD)	17.5 (1.5)	17.5 (1.3)	0.701	0.019 (0.701)
Father’s education, %				
Secondary or less	22 (12.4)	155 (87.6)	0.604	-0.026 (0.605)
College and above	24 (10.8)	199 (89.2)		
Mother’s education, %				
Secondary or less	25 (13.0)	167 (87.0)	0.360	-0.046 (0.361)
College and above	21 (10.1)	187 (89.9)		
Family income, %				
15,000 SAR or less	20 (8.1)	226 (91.9)	0.008	0.134 (0.008)
More than 15,000 SAR	26 (16.9)	128 (83.1)		
Father’s employment, %				
Employed	21 (13.5)	134 (86.5)	0.307	-0.051 (0.308)
Unemployed	25 (10.2)	220 (89.8)		
Mother’s employment, %				
Employed	33 (13.0)	221 (87.0)	0.217	-0.062 (0.218)
Unemployed	13 (8.9)	133 (91.1)		
Number of siblings, %				
Less than five	6 (7.9)	70 (92.1)	0.274	0.055 (0.275)
Five and more	40 (12.3)	284 (87.7)		
Grade level				
Good	5 (23.8)	16 (76.2)	0.029	-0.109 (0.029)
Very good	13 (14.6)	76 (85.4)		
Excellent	28 (9.7)	262 (90.3)		
School level				
First	7 (20.0)	28 (80.0)	0.692	0.020 (0.693)
Second	3 (3.3)	89 (96.7)		
Third	36 (13.2)	237 (86.8)		

Substance abuse and illicit drugs

A total of 38 students (9.5%) claimed that they had attempted the use of drugs, such as cocaine, heroin, amphetamine, marijuana, and other illegal drugs. The most commonly abused substance was marijuana (n=16, 4.0%). Of the 38 students, 14 (36.8%) began using illegal substances at 14-16 years old, whereas 16 (33.3%) began using these substances after age 20. Eighteen of the 38 students (47.4%) could obtain illicit drugs from friends, 10 (26.3%) by themselves, and 10 (26.3%) either by themselves or through friends. Fourteen of the 38 students (36.8%) took these illicit drugs once per month, while 13 (34.2%) and seven (18.4%) took these drugs on a weekly basis and daily basis, respectively.

There were significantly more students who belonged to families with higher monthly income who had attempted the use of drugs and illicit substances than students in lower family income brackets (p=0.050). Furthermore, there were more students who had less than five siblings attempted use of illicit substances than those who had more than five siblings (p<0.001). Few students who used illicit substances achieved excellent grades (*p=0.049). The use of illegal drugs and illicit substances was significantly positively correlated with higher monthly family income (r=0.094, p=0.050) and significantly negatively correlated with number of siblings (r=-0.191, p<0.001) and grades (r=-0.098, p=0.050) (Table 4).

**Table 4:** Attempted/used illegal drugs or illicit substances versus who did not attempt to use according to demographic characteristics

Variables	Drug and illicit substances use, n=38	Not attempted to use drugs and illicit substances, n=362	P-values	Correlation (p-values)
Age in years, mean (SD)	17.5 (1.5)	17.4 (1.3)	0.398	0.00 (0.995)
Father’s education, %				
Secondary or less	18 (10.2)	159 (89.8)	0.684	-0.020 (0.685)
College and above	20 (9.0)	203 (91.0)		
Mother’s education, %				
Secondary or less	22 (11.5)	170 (88.5)	0.199	-0.064 (0.200)
College and above	16 (7.7)	192 (92.3)		
Family income, %				
15,000 SAR or less	18 (7.3)	228 (92.7)	0.050	0.094 (0.050)
More than 15,000 SAR	20 (13.0)	134 (87.0)		
Father’s employment, %				
Employed	19 (12.3)	136 (87.7)	0.135	-0.075 (0.135)
Unemployed	19 (7.8)	226 (92.2)		
Mother’s employment, %				
Employed	27 (10.6)	227 (89.4)	0.309	-0.051 (0.311)
Unemployed	11 (7.5)	135 (92.5)		
Number of siblings, %				
Less than five	16 (21.1)	60 (78.9)	<0.001	-0.191 (<0.001)
Five and more	22 (6.8)	302 (93.2)		
Grade level				
Good	4 (19.0)	17 (81.0)	0.049	-0.098 (0.050)
Very good	11 (12.4)	78 (87.6)		
Excellent	23 (7.9)	267 (92.1)		
School level				
First	5 (14.3)	30 (85.7)	0.518	-0.021 (0.671)
Second	7 (7.6)	85 (92.4)		
Third	26 (9.5)	247 (90.5)		

Reasons students began smoking, drinking, or taking illicit drugs and substances

The most common reasons for smoking, drinking, or taking elicits drugs and substances were loneliness (n=39, 9.8%) and seeking happiness (n=39, 9.8%). Other reasons included curiosity (n=38, 9.5%), influence of peers and friends (n=33, 8.3%), and to forget problems (n=17, 4.3%). 

Knowledge and opinions of smoking, drinking alcohol, and substance abuse

Most students believed that a person who smoked, drank alcohol, and took drugs always had unsatisfactory health (73.8%), developed respiratory disease (64.3%), developed heart disease (65.0%), spent more money (75.0%), dropped out of school (60.8%), and always ran out of money (66.7%). Furthermore, a large proportion of students believed that smoking, alcohol, and drug abuse were against the norms of society (76.0%). They also believed that people do not like the company of people who smoke, drink alcohol, use illicit substances (69.0%), and these people are a burden to society (69.8%). Seventy-two percent of the students believed that smoking, alcohol, and substance abuse are dangerous to their health. However, there were divided views on whether the family of a person who smoked, drank alcohol, and took drugs always experienced poverty, where 154 (38.5%) were undecided. Students do not easily trust a person that uses illicit drugs compared to people who drink alcohol and those who smoke (79.3% versus 68.3% versus 37.8%, respectively) (Table 5).

**Table 5:** Knowledge and opinions of male high school students with regard to smoking, drinking alcohol, and substance abuse.

Questions	Strongly agree/agree	Neutral	Strongly disagree/disagree
Smoking, alcohol, and substance abuse are not dangerous to my health	76 (19.0%)	36 (9.0%)	288 (72.0%)
A person who smokes, drinks alcohol, and takes drugs always has unsatisfactory health	295 (73.8%)	48 (12.0%)	57 (14.2%)
A person who smokes, drinks alcohol, and takes drugs develops respiratory disease	257 (64.3%)	96 (24.0%)	47 (11.7%)
A person who smokes, drinks alcohol, and takes drugs develops heart disease	260 (65.0%)	89 (22.2%)	51 (12.8%)
A person who smokes, drinks alcohol, and takes drugs spends more money	300 (75.0%)	40 (10.0%)	60 (15.0%)
A person who smokes, drinks alcohol, and takes drugs drops out of school	243 (60.8%)	77 (19.2%)	80 (20.0%)
A person who smokes, drinks alcohol, and takes drugs always runs out of money	267 (66.7%)	70 (17.5%)	63 (15.8%)
The family of the person who smokes, drinks alcohol, and takes drugs always experiences poverty	131 (32.8%)	154 (38.5%)	115 (28.7%)
Smoking, alcohol, and drug abuse are against the norms of society	304 (76.0%)	40 (10.0%)	56 (14.0%)
The family of the person who smokes, drinks alcohol, and takes drugs is always denigrated by others	206 (51.5%)	129 (32.2%)	65 (16.3%)
People do not like the company of people who smoke, drink alcohol, and take drugs	276 (69.0%)	63 (15.8%)	61 (15.2%)
A person who smokes, drinks alcohol, and takes drugs is a burden to society	279 (69.8%)	54 (13.5%)	67 (16.7%)
People do not easily trust a person that smokes	151 (37.8%)	103 (27.7%)	146 (36.5%)
People do not easily trust a person that drinks alcohol	273 (68.3%)	72 (18.0%)	55 (13.7%)
People do not easily trust a person that takes illicit drugs	317 (79.3%)	41 (10.2%)	42 (10.5%)

Attitudes toward smoking, alcohol drinking, and use of drugs and illicit substances

Most students (75.5%) would refuse an offer of cigarettes, alcohol, or drugs; however, 70 students (17.5%) would smoke, drink, or use drugs and illicit substances owing to curiosity. Many students (n=274, 68.5%) would resist invitations, 280 (70.0%) would avoid places that sell these items, and 227 (56.8%) engage in sports and other activities or are involved in social work (n=205, 51.2%) to avoid these vices. Many students denigrated people who use illicit drugs more than those who drink alcohol and smoke (66.3% vs. 55.0% versus 35.8%, respectively). Seven of 10 male students believed that occasional smoking, alcohol drinking, and illicit drug use are extremely harmful (70.8%) and can kill a person (75.8%). However, only eight of the 10 students (81.0%) believed that smoking, alcohol drinking, and substance abuse are dangerous to their health (Table 6).

**Table 6:** Attitudes of male high school students toward smoking, alcohol drinking, and the use of drugs and illicit substances

Questions	Strongly agree/agree	Neutral	Strongly disagree/disagree
I will refuse an offer of cigarette, alcohol, or drugs	302 (75.5%)	40 (10.0%)	58 (14.5%)
I will try at least once to smoke, drink alcohol, or take drugs out of curiosity	70 (17.5%)	62 (15.5%)	268 (67.0%)
I can resist the invitation from my peers to try smoking, alcohol, and drug use	274 (68.5%)	55 (13.8%)	71 (17.7%)
I avoid places that sell illicit substances and smoke	280 (70.0%)	64 (16.0%)	56 (14.0%)
I practice sports and other activities to avoid these vices	227 (56.8%)	84 (21.0%)	89 (22.2%)
I participate in social work to help these people	205 (51.2%)	123 (30.8%)	72 (18.0%)
I denigrate people who smoke	143 (35.8%)	112 (28.0%)	145 (36.2%)
I denigrate people who drink alcohol	220 (55.0%)	101 (25.2%)	79 (19.8%)
I denigrate people who use illicit drugs	265 (66.3%)	55 (13.7%)	80 (20.0%)
I believe that occasional smoking, alcohol drinking, and illicit drug use are extremely harmful	283 (70.8%)	53 (13.2%)	64 (16.0%)
I believe that taking illicit drugs will kill a person	303 (75.8%)	55 (13.7%)	42 (10.5%)
Smoking, alcohol, and substance abuse are not dangerous to my health	76 (19.0%)	0	324 (81.0%)

## Discussion

This study aimed to determine the prevalence rates of smoking, alcohol consumption, and use of illicit substances among high school students. Smoking, alcohol consumption, and the use of illicit substances have long been reported; however, worldwide reports still show a high prevalence of such practices in more than 40% of high school students [2-5]. Since its inclusion in the WHO Framework Convention on Tobacco Control in 2005, Saudi Arabia has legislated strict laws on advertising, promotion, and sponsorship to restrict sales through the Royal Decree No. 56 or the so-called “Anti-Smoking Law” [10]. Similarly, alcohol, illegal drugs, and the use of illicit substances remain taboo.

Smoking

The prevalence of smoking in this study was 27.8%. This prevalence is lower than the 37.1% found among male secondary students in Jeddah, as reported by Fida and Abdelmoneim in 2013, and 40.8% reported by Albangy et al. in 2019; however, it is higher than the 15.17% prevalence found among intermediate and secondary school students in Madina, Saudi Arabia in 2013 [11-13]. Similar to previous studies, having friends and peers who smoke has a significant influence on the smoking status of students [11-13]. One of the highlights of this study is the ability of 76.1% of the students who smoke to purchase cigarettes from nearby stores. This occurrence is despite the strict implementation of cigarette sales among students in Saudi Arabia. The magnitude of smoking is reflected by the number of respondents who smoked daily (89.6%). Further, 58.9% of these students began smoking before the age 15 years old. Such a finding is alarming as smoking at an early age is correlated with lifelong smoking, which makes it more difficult for them to quit when they are older [14]. Several other studies on smoking among students conducted in Saudi Arabia had comparable prevalence rates: AlHassa (28.1%) [15], and 28.6% from the National Guard study in 2009 [16]. 

This study revealed no significant correlation between smoking and school level, age, grades, parents’ educational level, parents' employment, number of siblings, and income. The significant effects of the parent’s level of education, parent employment, and monthly family income were not found in this study as most parents were educated and had a high monthly income. Students who had better-educated parents and with good family income were better and more effective at school, as these factors markedly influenced student behavior [17]. The strong effect of friends who smoke, parents who smoke, and siblings who smoke were revealed in previous studies. Early smoking, particularly among teenagers and adolescents, was explained by the presence of siblings and peer smoking [18]. A study showed that the prevalence of teenage and adolescent smoking increased from 18% (where no parent or sibling smokes) to 31% when at least one sibling smoke, and to as much as 41% when a parent and a sibling smoke [17,18].

Drinking alcohol

The prevalence of alcohol consumption among the respondents was 11.5%. This prevalence is higher than that of previous studies where 9.3% of male secondary school students drank alcohol [7]. In a study conducted in Jeddah in 2015, 2.6% of students drank alcohol [19]. More than half of the 46 students who consumed alcohol began drinking before age 20. The age at onset of alcohol consumption was reported to have a significant negative correlation with the severity of alcoholism in adulthood and postponing the use of alcohol until age 25 may be a good strategy to prevent severe alcoholism in adulthood [20].

A positive association was found between alcohol intake and higher family income. Further, students who drank alcohol had a significantly lower performance at school. Whether these students had siblings and parents who drank alcohol was not investigated. Studies have shown that alcohol intake among parents and siblings predisposes their son or younger brothers to alcoholism, besides the heightened genetic risk for the development of alcohol abuse [21].

Use of drugs and illicit substances

The prevalence of drug abuse and use of illicit substances in this study was 9.5%. This percentage is slightly higher than the 8.8% reported by Musa in 2016 [7]. Although both studies revealed the use of cannabis (marijuana), this study revealed many other more dangerous substances, including cocaine and heroin. Our findings also confirm those of previous studies that young men abuse drugs and take illicit substances because of peer pressure [22], where 47.4% of our respondents could obtain these drugs and substances from friends, in contrast to smoking products and alcohol. Similar to other studies, 14 to 16 years were identified as the most volatile ages. A study on drug abuse and drug dependents admitted to a health institution in Riyadh showed an age range of 11-21 years old, with predominantly (53.3%) male high school graduates [22]. Similar to findings regarding smoking and alcohol intake, the use of drugs and illicit substances was associated with poor school performance, higher monthly income families and a lesser number of siblings. Such finding might be due to the fact that drug users and substance abusers have a capacity to buy and procure these substances and greater tendency to hide their vices from their parents and siblings, but usually carry out these vices with friends and peers and also may be due to the lower sample size of people who take these illicit substances.

Reasons for smoking, alcohol intake, and drug abuse

Loneliness and wanting to be happy and curiosity were the main reasons for smoking, drinking alcohol, and abusing drugs in this survey. Such finding is not surprising, as many reports have suggested that loneliness is particularly familiar among adolescents and young adults that turn to these vices [23]. Adolescents and young adults face peer pressure and experience a feeling of connection and social acceptance. Teenagers, who usually connect with peers who smoke, drink alcohol and use illicit substances, are lured not just by peer pressure or sense of belongingness but also by curiosity. These in turn make these vices easily accessible to them. Thus, during these years, they require social support as they are experiencing social changes and coping with social stress, including romantic relationships and relationships with family and friends [24]. Very few adolescents can control their emotions to cope with stress, anxiety, and depression, which may lead to drug use that can be heightened by the influence of peers who are also drug users [25].

Knowledge, opinions, and attitudes toward smoking, drinking alcohol, and substance abuse

Despite the high prevalence of smoking, alcohol, and drug use among our respondents, seven of 10 believed that smoking, drinking alcohol, and drug abuse are not good for their health. Most students perceived that smoking, drinking alcohol, and drug abuse were a burden to society. These perception and knowledge echo those of previous studies where adolescents were knowledgeable about the aspects of smoking and alcoholism, but less knowledgeable about substance abuse [26,27]. Despite their high knowledge and negative perception of smoking, drinking alcohol, and drug abuse, there is still a high prevalence of smokers, alcohol drinkers, and drug users, similar to other reports [28]. Further, despite their knowledge, healthy behaviors are not practiced, and unhealthy vices are not curbed.

Approximately 20%-25% of our respondents believed that these unhealthy vices are not harmful to their health. In fact, only seven of 10 will resist taking or using these substances. A study revealed that knowledge of the hazards of smoking, and even alcohol and illicit substances, does not influence smoking and the use of illicit substance [29]. Although some studies have implicated indicators for the use of these drugs, tobacco, and alcohol, which include family income, wealth, and parental education, these factors were not observed in this study [30].

Limitations of the study

The nature of the survey served as a limitation. The responses were self-reported by students; thus, the possibility of underreporting must be considered. We also failed to extract information on the family structure of smoking, alcoholism, and drug use by other family members to determine its association with the use of these substances by students. On the lighter side, we demonstrated the prevalence of smoking, alcohol intake, and substance abuse that can be employed as a basis by our group as well as other researchers for further studies on this subject matter.

## Conclusions

Among the male high school population evaluated herein, the prevalence of smoking was quite high (27.8%). Further, the prevalence of alcohol intake was 11.5%, while that of drug abuse or use of illicit substances was 9.5%. Students began smoking, drinking alcohol, and abusing drugs at an early age, which may be influenced by friends and peers or siblings. These students procure these substances by themselves while some receive help from friends, particularly for alcohol. These practices affect the performance of students at school. Although many students know the harmful effects of smoking, alcohol intake, and substance abuse, some students had an opposing perspective. Health authorities need to educate these students and institute structural and emotional support for students who are in these vices to mitigate misuse, long-term use, and addiction.

## References

[1] US Department of Health and Human Services (2014). The health consequences of smoking −50 years of progress. https://www.cdc.gov/tobacco/data_statistics/sgr/50th-anniversary/index.htm.

[2] Arrazola RA, Singh T, Corey CG (2015). Tobacco use among middle and high school students - United States, 2011-2014. MMWR Morb Mortal Wkly Rep.

[3] Singh T, Arrazola RA, Corey CG, Husten CG, Neff LJ, Homa DM, King BA (2016). Tobacco use among middle and high school students - United States, 2011-2015. MMWR Morb Mortal Wkly Rep.

[4] Jamal A, Gentzke A, Hu SS, Cullen KA, Apelberg BJ, Homa DM, King BA (2017). Tobacco use among middle and high school students - United States, 2011-2016. MMWR Morb Mortal Wkly Rep.

[5] Moh'd Al-Mulla A, Abdou Helmy S, Al-Lawati J (2008). Prevalence of tobacco use among students aged 13-15 years in Health Ministers' Council/Gulf Cooperation Council Member States, 2001-2004. J Sch Health.

[6] Al Agili DE, Park HK (2012). The prevalence and determinants of tobacco use among adolescents in Saudi Arabia. J Sch Health.

[7] Al-Musa HM, Al-Montashri SD (2016). Substance abuse among male secondary school students in Abha City, Saudi Arabia: Prevalence and associated factors. Biomed Res.

[8] Gaffar AM, Alsanosy RM, Mahfouz MS (2013). Sociodemographic factors associated with tobacco smoking among intermediate and secondary school students in Jazan Region of Saudi Arabia. Subst Abus.

[9] Siddiqui AF, Salim AM (2016). Awareness of substance use and its associated factors in young Saudi students. JMAS.

[10] (2020). Tobacco control laws: Saudi Arabia. https://www.tobaccocontrollaws.org/legislation/country/saudi-arabia/summary.

[11] Fida HR, Abdelmoneim I (2013). Prevalence of smoking among male secondary school students in Jeddah, Saudi Arabia. J Family Community Med.

[12] Albangy FH, Mohamed AE, Hammad SM (2019). Prevalence of smoking among male secondary school students in Arar City, Saudi Arabia. Pan Afr Med J.

[13] Al-Zalabani A, Kasim K (2015). Prevalence and predictors of adolescents' cigarette smoking in Madinah, Saudi Arabia: a school-based cross-sectional study. BMC Public Health.

[14] Warren CW, Jones NR, Eriksen MP, Asma S (2006). Pattern of global tobacco use in young people and implications for further chronic diseases burden in adults. Lancet.

[15] Al Mohamed HI, Amin TT (2010). Pattern and prevalence of smoking among students at King Faisal university, Al Hassa, Saudi Arabia. EMHJ-eastern Mediterranean Health. Eastern Mediterranean Health J.

[16] Al Nohair SF (2011). Prevalence of smoking and its related behaviors and beliefs among secondary school students in Riyadh, Saudi Arabia. Int J Health Sci (Qassim).

[17] Zaloudíková I, Hrubá D, Samara I (2012). Parental education and family status--association with children's cigarette smoking. Cent Eur J Public Health.

[18] Kelly AB, O'Flaherty M, Connor JP, Homel R, Toumbourou JW, Patton GC, Williams J (2011). The influence of parents, siblings and peers on pre- and early-teen smoking: a multilevel model. Drug Alcohol Rev.

[19] Beaver KM, Al-Ghamdi MS, Kobeisy AN, Alqurashi FH, Schwartz JA, Connolly EJ, Gajos JM (2016). The effects of low self-control and delinquent peers on alcohol, tobacco, and drug use in a sample of Saudi Arabian youth. Int J Offender Ther Comp Criminol.

[20] Johnson PR, Banu S, Ashok MV (2010). Severity of alcoholism in Indian males: correlation with age of onset and family history of alcoholism. Indian J Psychiatry.

[21] Njoroge BN (2020). Influence of parental alcoholism on academic performance of secondary school students in Kandara sub county, Muranga County-Kenya (doctoral dissertation, Pan Africa Christian University). Springer Link J.

[22] Al-Jerani FM, Al-Basry EA, Aldawood H, Almudhry ZA, Alshammari NM, Busaleh H (2017). Substance abuse among Saudi population. IJMDC.

[23] Lamis DA, Ballard ED, Patel AB (2014). Loneliness and suicidal ideation in drug-using college students. Suicide Life Threat Behav.

[24] Lee CY, Goldstein SE (2016). Loneliness, stress, and social support in young adulthood: does the source of support matter?. J Youth Adolesc.

[25] Spooner C, Hetherington K (2005). Social determinants of drug use. NDARC.

[26] Haddad L, Shotar A, Umlauf M, Al-Zyoud S (2010). Knowledge of substance abuse among high school students in Jordan. J Transcult Nurs.

[27] Martinotti G, Lupi M, Carlucci L (2015). Novel psychoactive substances: use and knowledge among adolescents and young adults in urban and rural areas. Hum Psychopharmacol.

[28] Malara B, Góra-Kupilas K, Jośko J, Malara P (2005). Evaluation of knowledge and health attitude towards cigarette smoking, alcohol and drugs use among students (Article in Polish). Przegl Lek.

[29] Ganley BJ, Rosario DI (2013). The smoking attitudes, knowledge, intent, and behaviors of adolescents and young adults: Implications for nursing practice. JNEP.

[30] Patrick ME, Wightman P, Schoeni RF, Schulenberg JE (2012). Socioeconomic status and substance use among young adults: a comparison across constructs and drugs. J Stud Alcohol Drugs.

